# Supplementary material for: Adaptive bias correction for improved subseasonal forecasting
Source: Nat Commun. 2023 Jun 15;14:3482. doi: 10.1038/s41467-023-38874-y (PMC10272189; doi:10.1038/s41467-023-38874-y)
Supplement: Supplementary file 1 — Supplementary Information [file 41467_2023_38874_MOESM1_ESM.pdf]

Supplementary Information for
Adaptive Bias Correction for Improved Subseasonal Forecasting

Soukayna Mouatadid[1], **Paulo Orenstein**[2], **Genevieve Flaspohler**[3,4,5], **Judah Cohen**[6,7], **Miruna Oprescu**[8], **Ernest Fraenkel**[9], **Lester Mackey**[10]

[1]Department of Computer Science, University of Toronto, Toronto, ON, Canada
[2]Instituto de Matemática Pura e Aplicada, Rio de Janeiro, Brazil
[3]*n*Line Inc., Berkeley, CA, USA
[4]Department of Electrical Engineering and Computer Science, Massachusetts Institute of Technology, Cambridge, MA, USA
[5]Department of Applied Ocean Science and Engineering, Woods Hole Oceanographic Institution, Falmouth, MA, USA
[6]Atmospheric and Environmental Research, Lexington, MA, USA
[7]Department of Civil and Environmental Engineering, Massachusetts Institute of Technology, Cambridge, MA, USA
[8]Department of Computer Science, Cornell University, Ithaca, NY, USA
[9]Department of Biological Engineering, Massachusetts Institute of Technology, Cambridge, MA, USA
[10]Microsoft Research New England, Cambridge, MA, USA

A Supplementary Figures

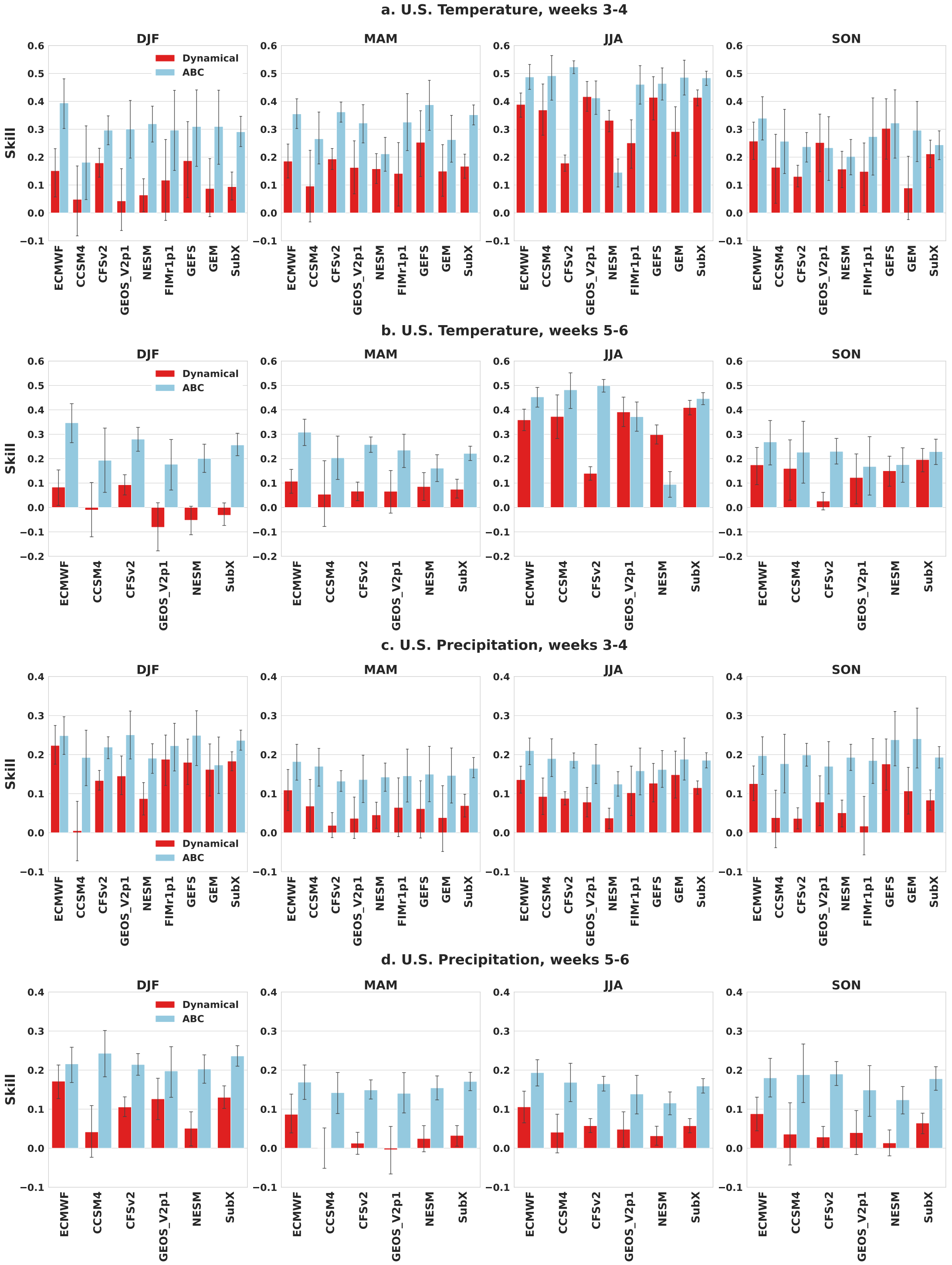

Figure S1: **Average forecast skill per season for dynamical models (red) and their ABC-corrected counterparts (blue).** For each forecasting task (**a, b, c, d**), skill is averaged across the contiguous U.S. and the years 2018–2021 with DJF = December, January, February; MAM = March, April, May; JJA = June, July, August; and SON = September, October, November. The error bars display 95% bootstrap confidence intervals. Models without forecast data for weeks 5-6 are omitted from the weeks 5-6 panels.

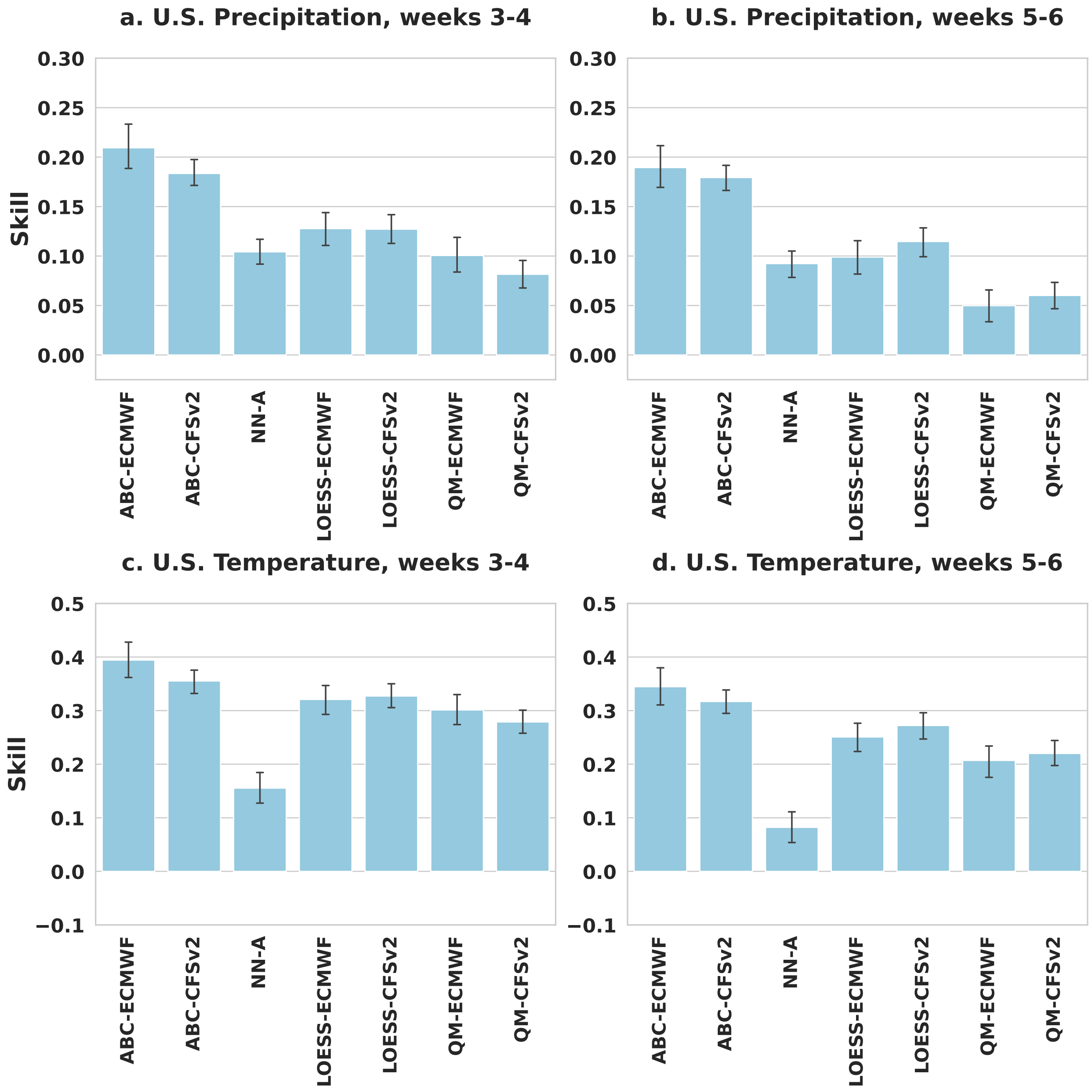

Figure S2: **Average forecast skill for adaptive bias correction (ABC) and baseline neural network (NN-A), locally estimated scatterplot smoothing (LOESS), and quantile mapping (QM) corrections of dynamical models.** For each forecasting task (**a, b, c, d**), skill is averaged across the contiguous U.S. and the years 2018–2021. The error bars display 95% bootstrap confidence intervals. The NN-A correction operates specifically on CFSv2 model inputs.

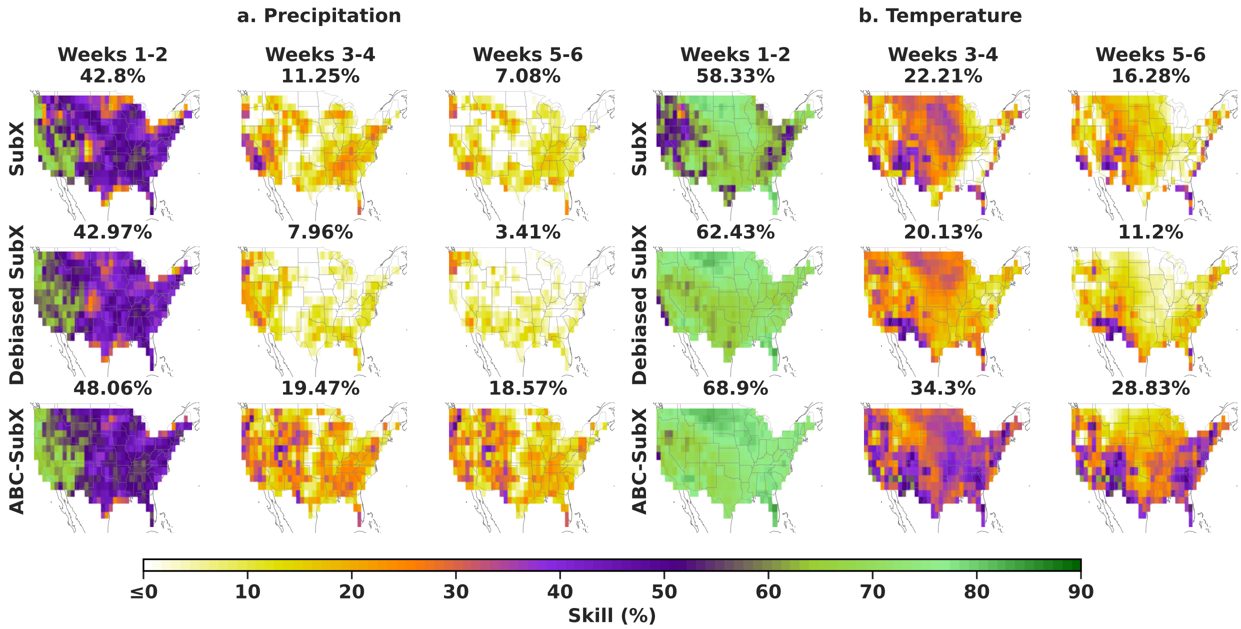

Figure S3: **Spatial skill distribution of SubX multimodel mean and adaptive bias corrections (ABC).** Across the contiguous U.S. and the years 2018–2021, SubX skill drops precipitously at subseasonal timescales (weeks 3-4 and 5-6), but ABC attenuates the degradation, boosting the skill of the SubX multimodel mean by 109-289% (over baseline skills of 0.07-0.22). Taking the same raw multimodel mean forecasts as input, ABC also provides consistent improvements over operational debiasing protocols, quadrupling the skill of the debiased SubX multimodel mean for precipitation (**a**) and doubling that of temperature (**b**). The average temporal skill over all forecast dates is displayed above each map.

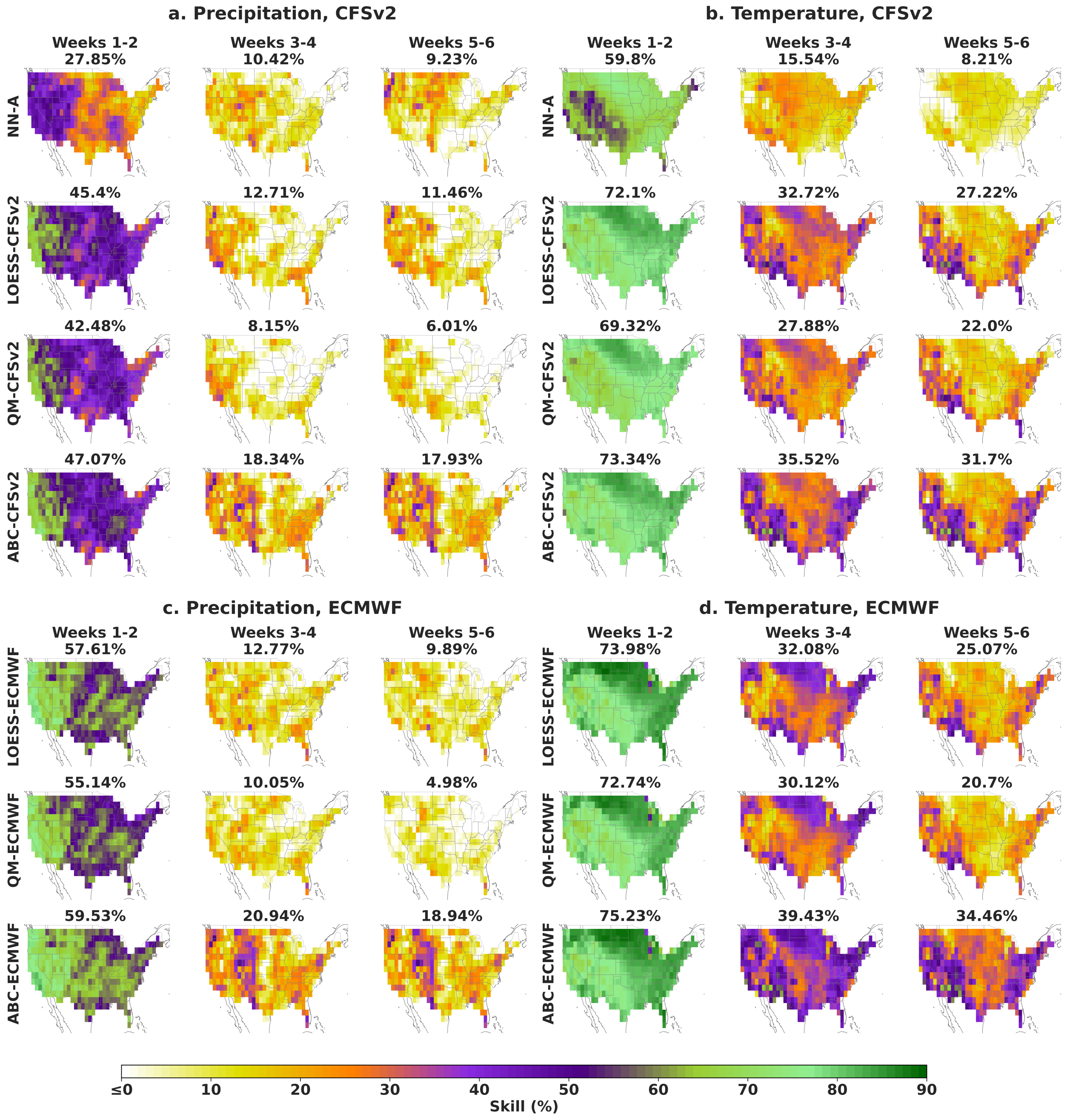

Figure S4: **Spatial distribution of model skill for adaptive bias correction (ABC) and baseline neural network (NN-A), locally estimated scatterplot smoothing (LOESS), and quantile mapping (QM) corrections of dynamical models.** For each grid point in the contiguous U.S., spatial skill is averaged over the years 2018–2021. The average temporal skill over all forecast dates is displayed above each precipitation (**a, c**) and temperature (**b, d**) map. The NN-A correction operates specifically on CFSv2 model inputs.

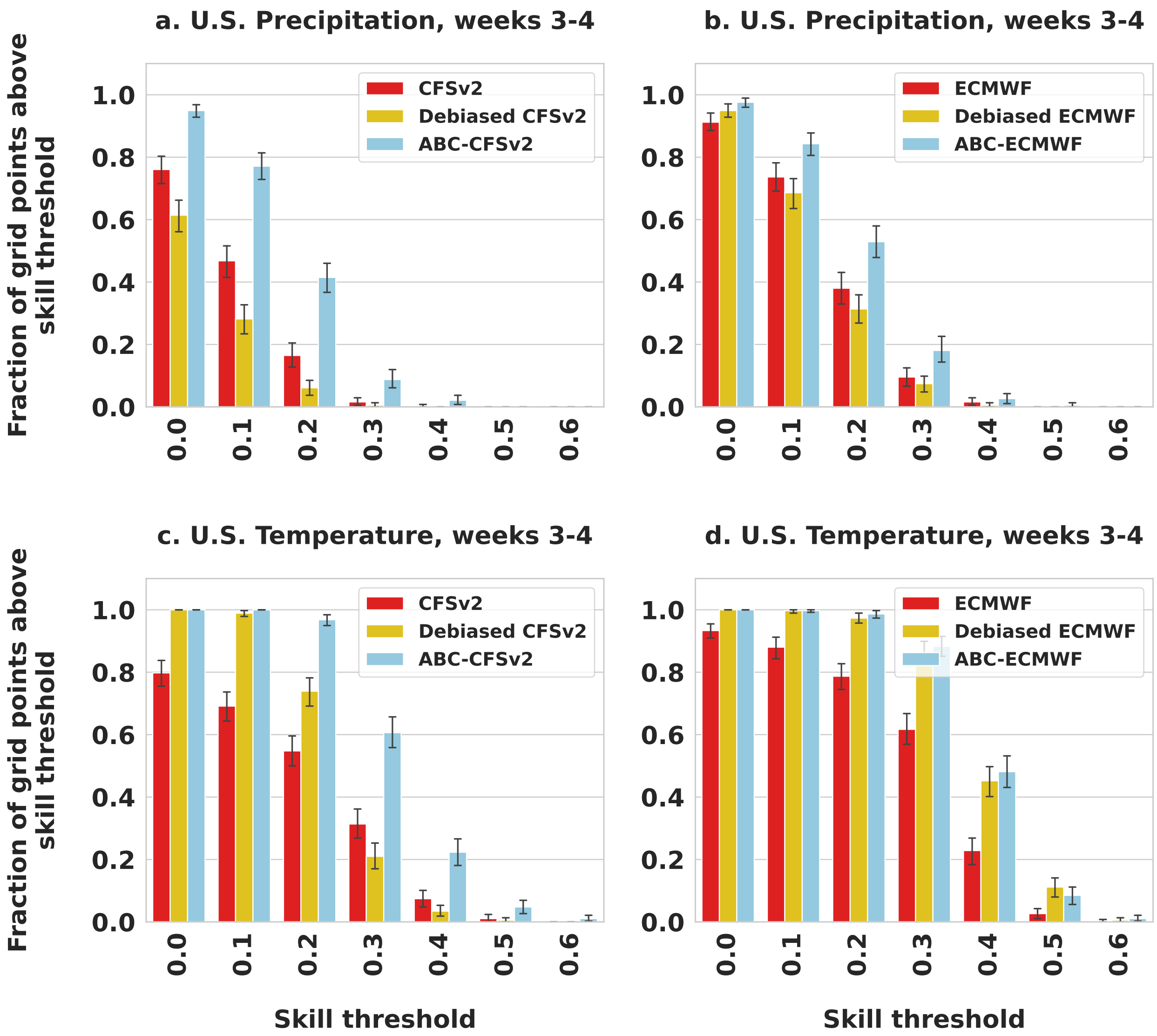

Figure S5: **Fraction of contiguous U.S. with 2018–2021 spatial skill above a given threshold.** For each forecasting task and dynamical model input (**a, b, c, d**), adaptive bias correction (ABC) consistently expands the geographic range of higher skill over raw and operationally-debiased dynamical models. The error bars display 95% bootstrap confidence intervals.

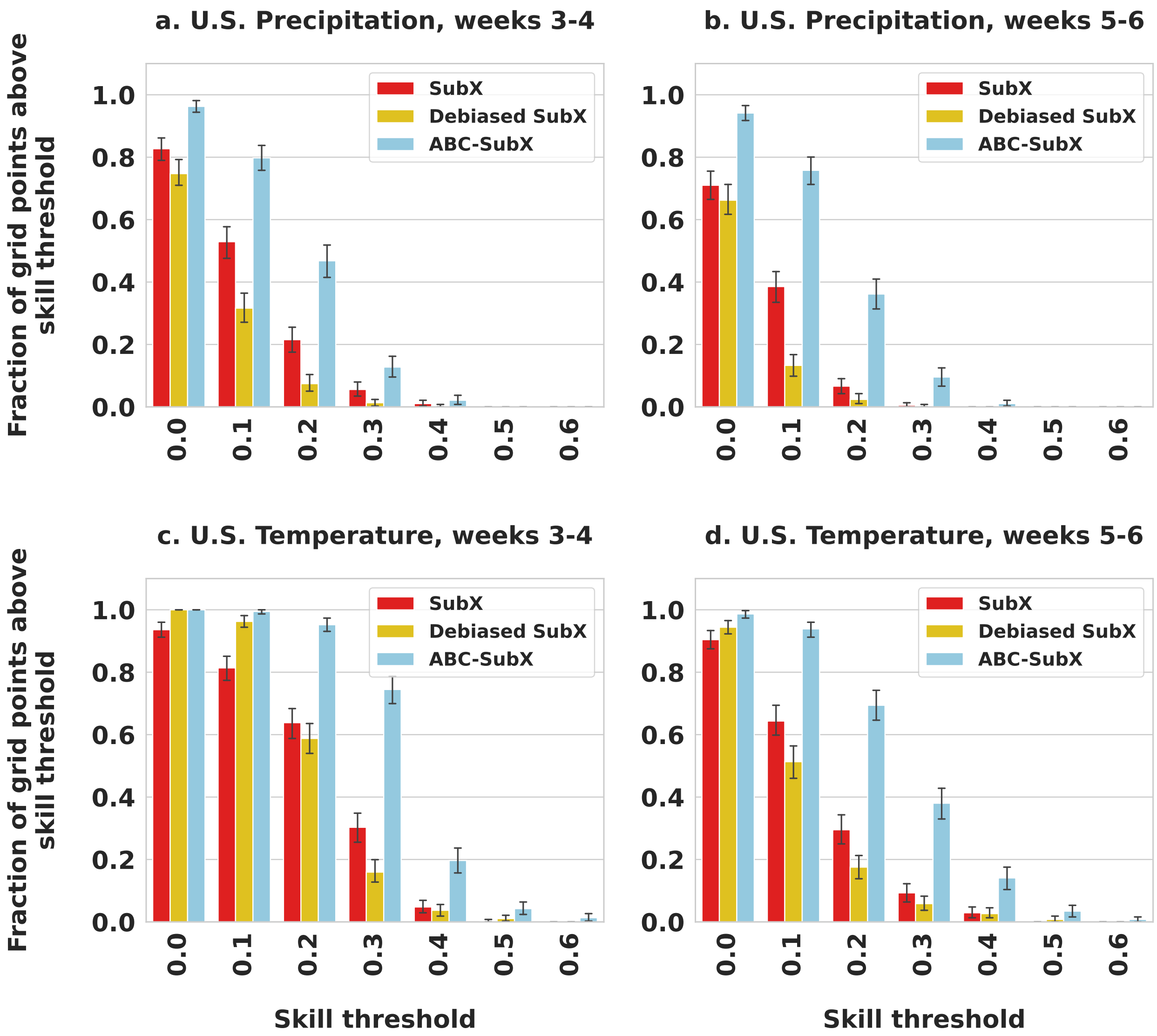

Figure S6: **Fraction of contiguous U.S. with 2018–2021 spatial skill above a given threshold.** For each forecasting task (**a, b, c, d**), adaptive bias correction (ABC) consistently expands the geographic range of higher skill over the raw and operationally-debiased SubX multimodel mean. The error bars display 95% bootstrap confidence intervals.

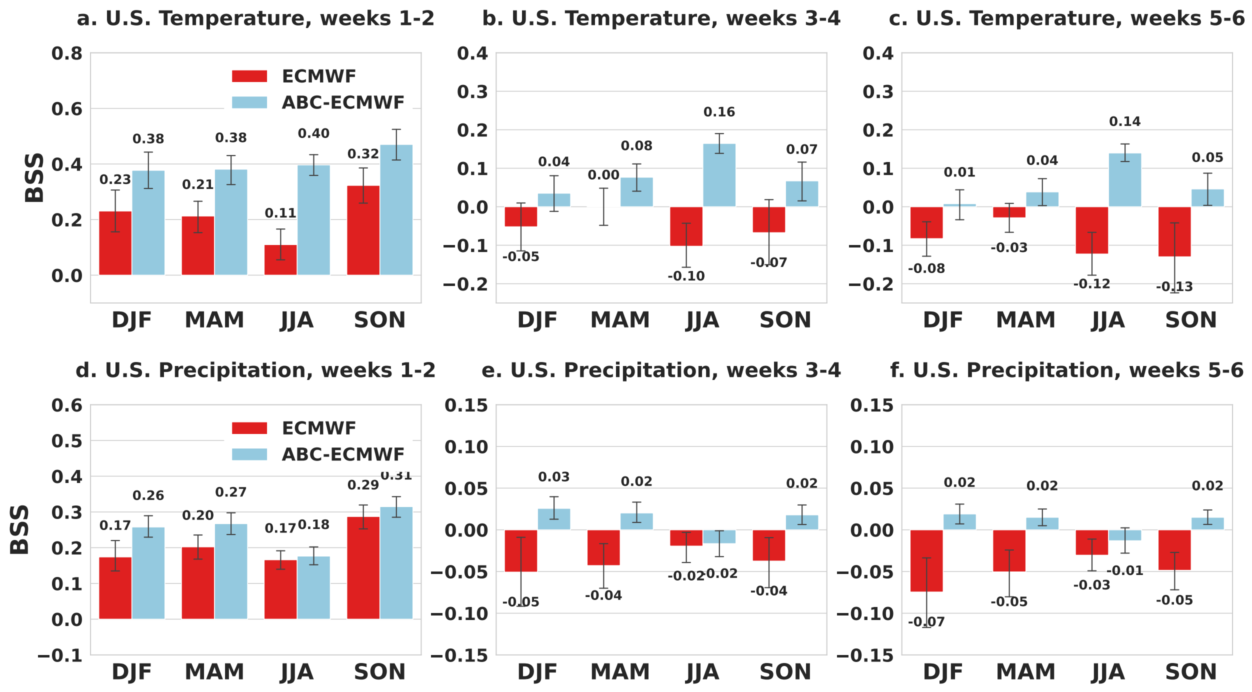

Figure S7: **Average Brier skill score (BSS) for above normal temperature or precipitation per season for ECMWF (red) and its adaptive bias correction (ABC) counterpart (blue).** Higher BSS indicates a more skillful probabilistic forecast. For each forecasting task (**a, b, c, d, e, f**), per-date BSS is computed across the contiguous U.S. and averaged over the years 2018–2021. Here, DJF = December, January, February; MAM = March, April, May; JJA = June, July, August; and SON = September, October, November. The error bars display 95% bootstrap confidence intervals.

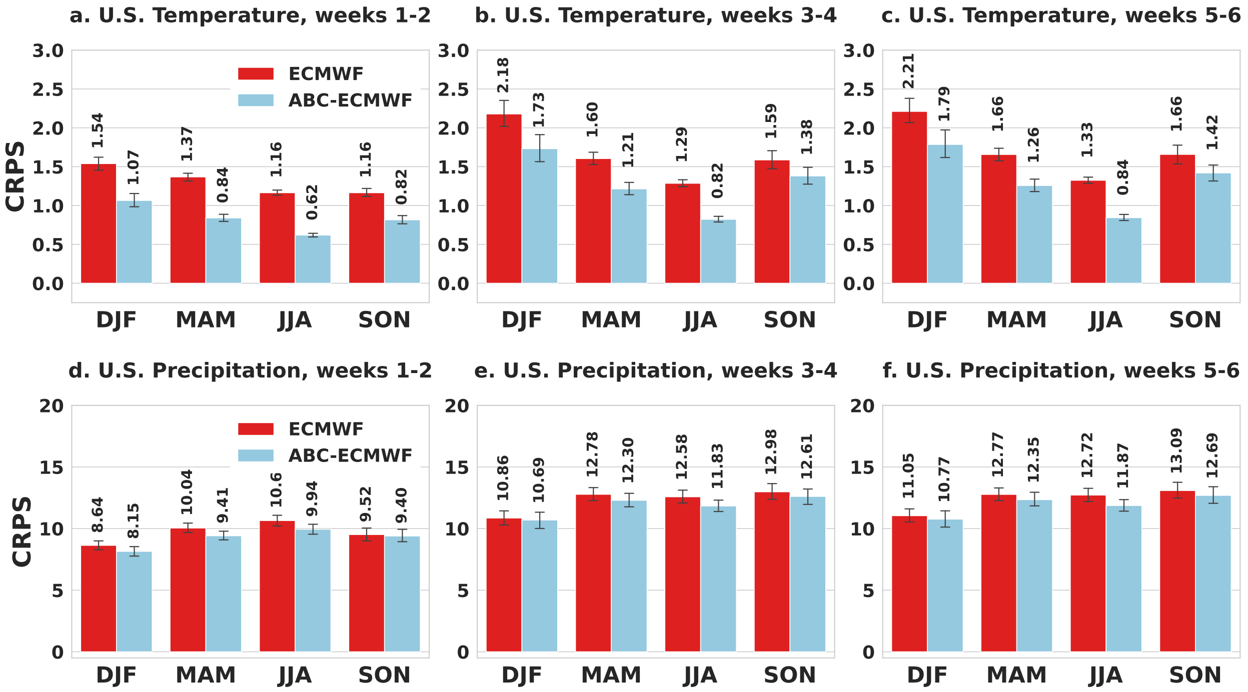

Figure S8: **Average continuous ranked probability score (CRPS) per season for ECMWF (red) and its adaptive bias correction (ABC) counterpart (blue).** Lower CRPS indicates a more accurate probabilistic forecast. For each forecasting task (**a, b, c, d, e, f**), per-date CRPS is computed across the contiguous U.S. and averaged over the years 2018–2021. Here, DJF = December, January, February; MAM = March, April, May; JJA = June, July, August; and SON = September, October, November. The error bars display 95% bootstrap confidence intervals.

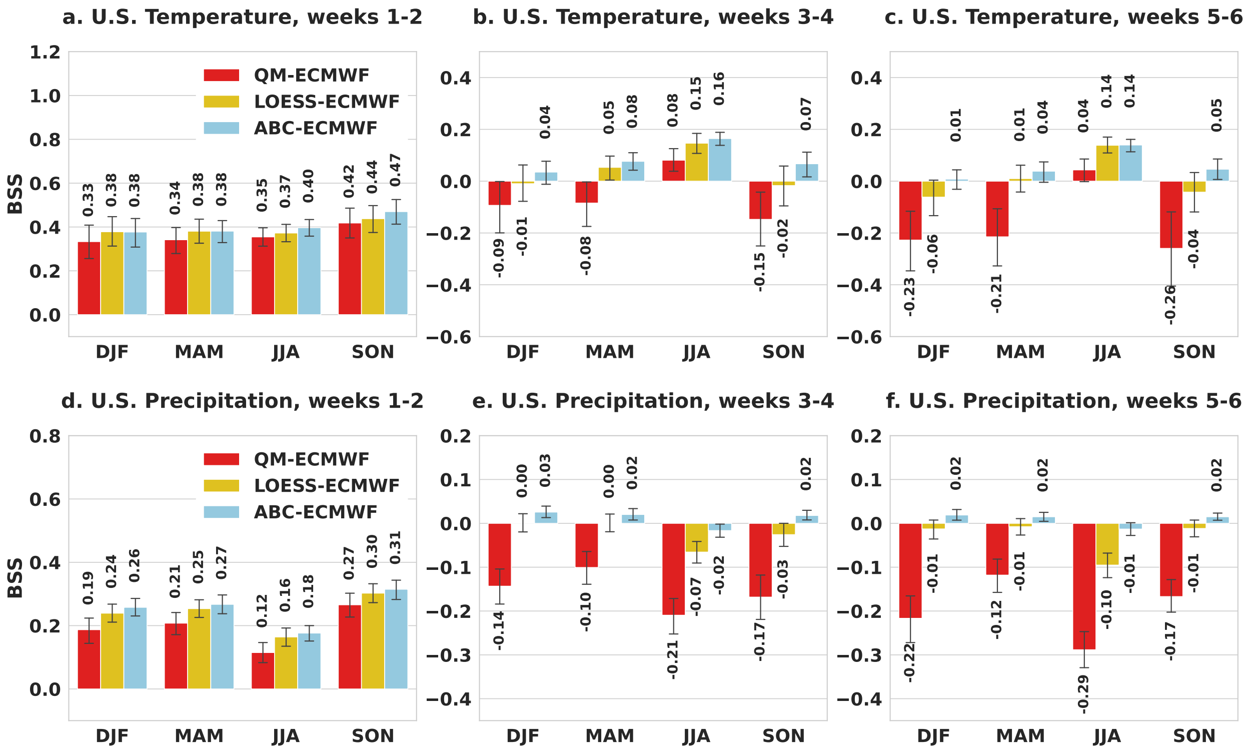

Figure S9: **Average Brier skill score (BSS) for above normal temperature or precipitation per season for adaptive bias correction (ABC) and baseline locally estimated scatterplot smoothing (LOESS) and quantile mapping (QM) corrections of ECMWF.** Higher BSS indicates a more skillful probabilistic forecast. For each forecasting task (**a, b, c, d, e, f**), per-date BSS is computed across the contiguous U.S. and averaged over the years 2018–2021. Here, DJF = December, January, February; MAM = March, April, May; JJA = June, July, August; and SON = September, October, November. The error bars display 95% bootstrap confidence intervals.

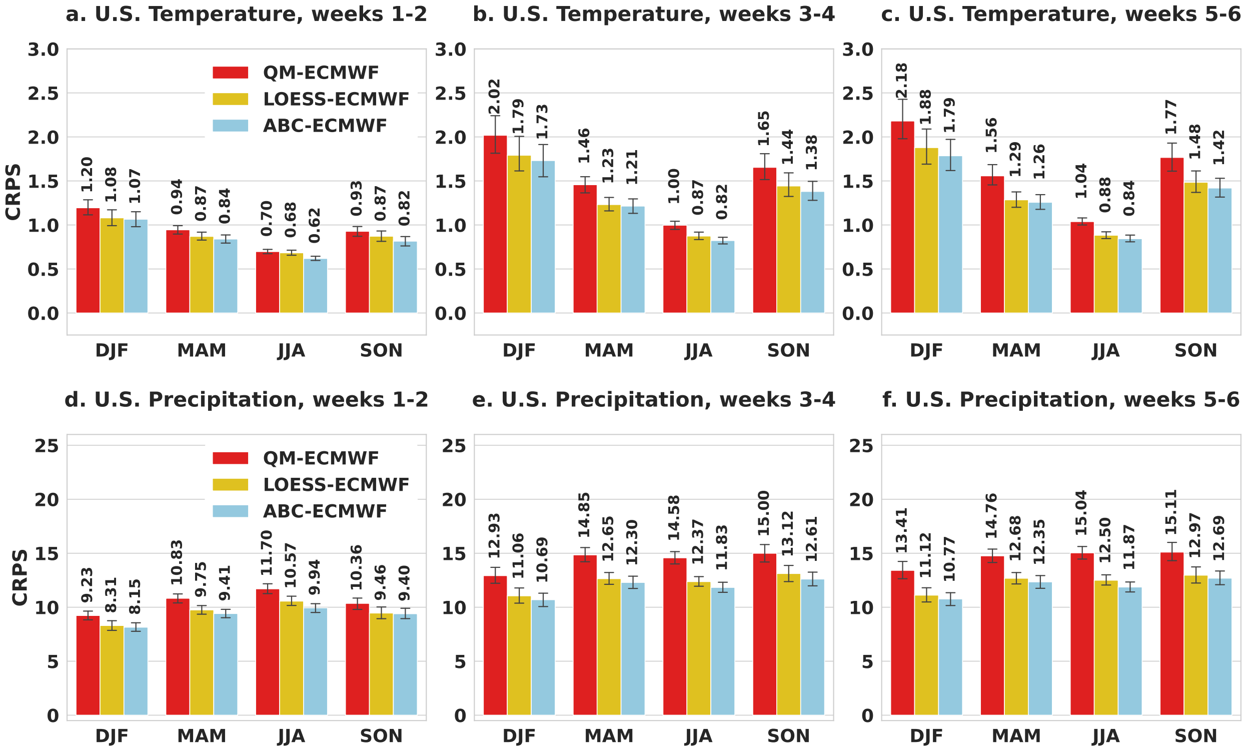

Figure S10: **Average continuous ranked probability score (CRPS) per season for adaptive bias correction (ABC) and baseline locally estimated scatterplot smoothing (LOESS) and quantile mapping (QM) corrections of ECMWF.** Lower CRPS indicates a more accurate probabilistic forecast. For each forecasting task (**a, b, c, d, e, f**), per-date CRPS is computed across the contiguous U.S. and averaged over the years 2018–2021. Here, DJF = December, January, February; MAM = March, April, May; JJA = June, July, August; and SON = September, October, November. The error bars display 95% bootstrap confidence intervals.

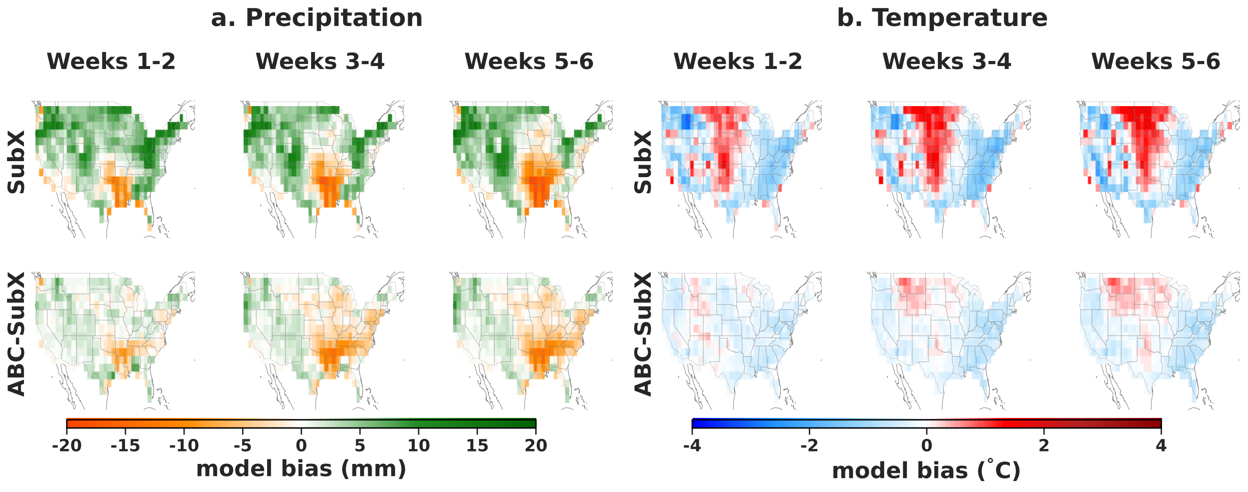

Figure S11: **Spatial distribution of model bias over the years 2018–2021.** Across the contiguous U.S., adaptive bias correction (ABC) reduces the systematic model bias of the SubX multimodel mean input for both precipitation (**a**) and temperature (**b**).

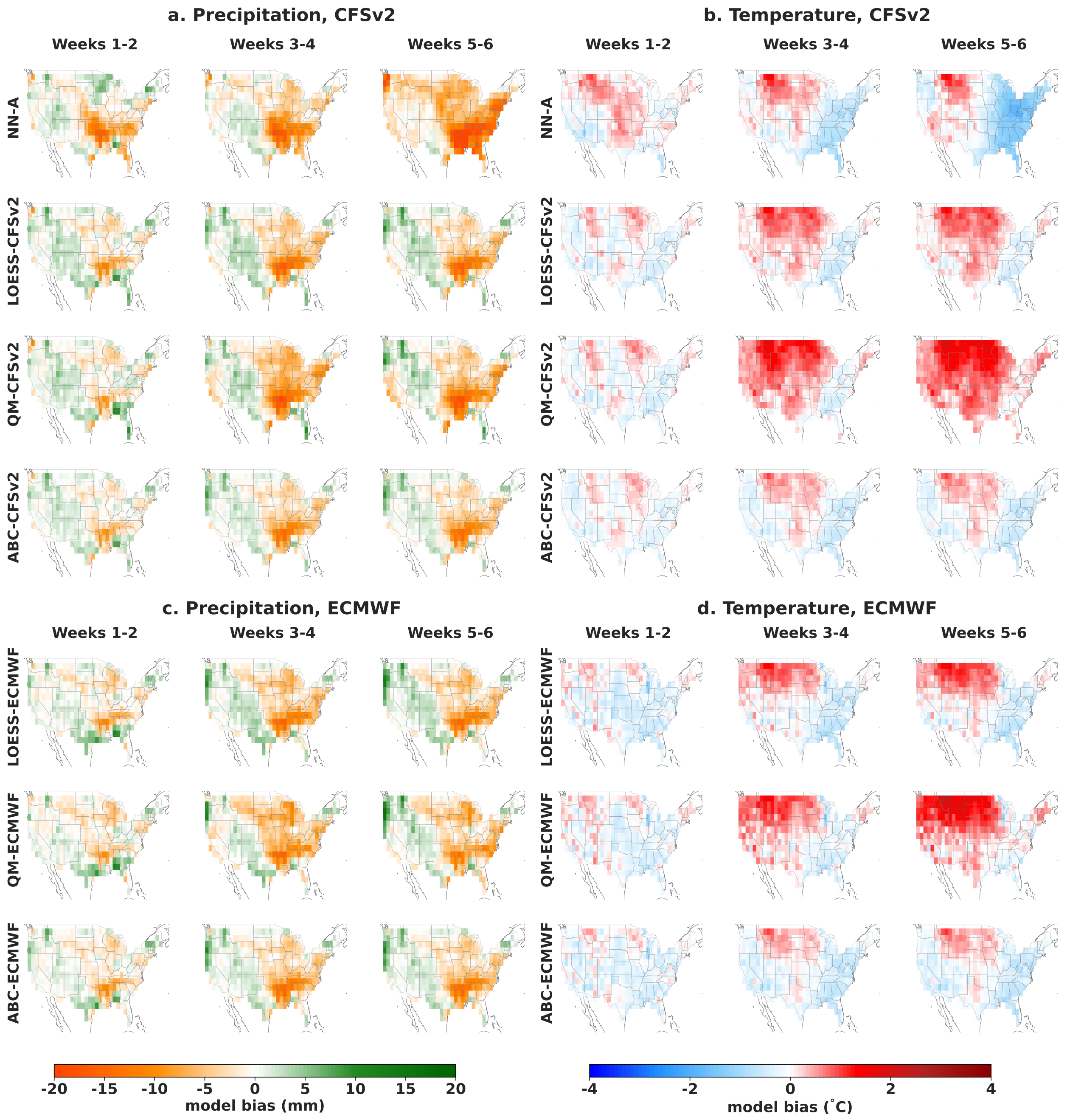

Figure S12: **Spatial distribution of model bias for adaptive bias correction (ABC) and baseline neural network (NN-A), locally estimated scatterplot smoothing (LOESS) and quantile mapping (QM) corrections of dynamical models.** For each grid point in the contiguous U.S., bias is computed over years 2018–2021 for both precipitation (**a**, **c**) and temperature (**b**, **d**). The NN-A correction operates specifically on CFSv2 model inputs.

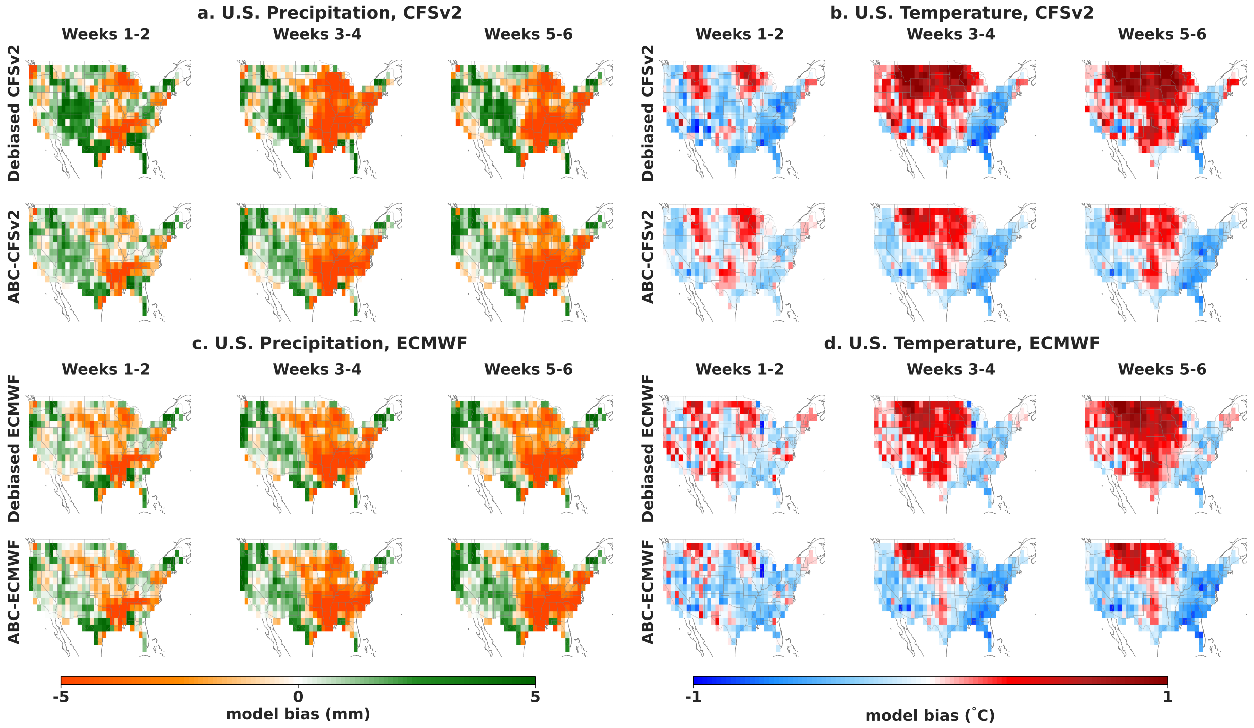

Figure S13: **Spatial distribution of model bias for adaptive bias correction (ABC) and operationally-debiased dynamical models.** For each grid point in the contiguous U.S., bias is computed over years 2018–2021 for both precipitation (**a**, **c**) and temperature (**b**, **d**).

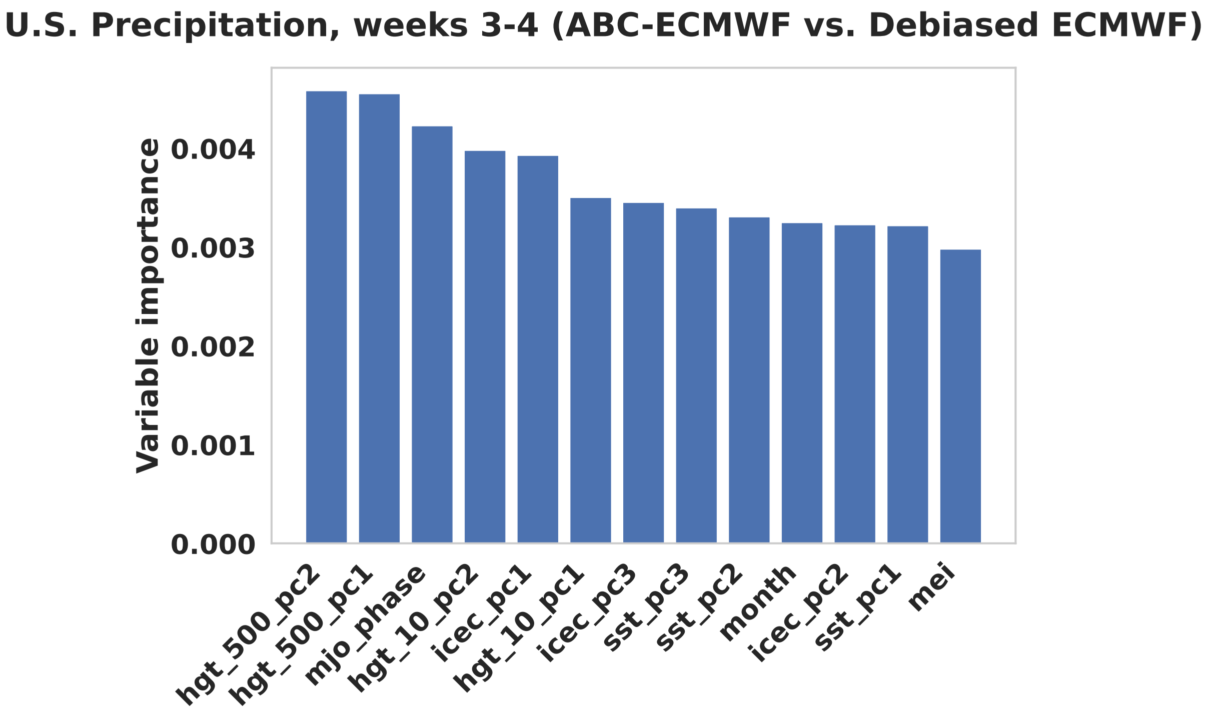

Figure S14: **Overall importance of explanatory variables in opportunistic adaptive bias correction (ABC) workflow.** The importance of each explanatory variable in explaining the weeks 3-4 precipitation skill improvement of ABC-ECMWF over debiased ECMWF is measured by Shapley effects. The explanatory variables considered are the first two principal components (PCs) of 500 hPa geopotential height (`hgt_500_pc1` and `hgt_500_pc2`), the first and second PC of 10 hPa geopotential height (`hgt_10_pc1` and `hgt_10_pc2`), the first three PCs of sea ice concentration (`icec_pc1`, `icec_pc2` and `icec_pc3`) and sea surface temperature (`sst_pc1`, `sst_pc2` and `sst_pc3`), the MJO phase (`mjo_phase`), the multivariate ENSO index (`mei`) and the month (`month`).

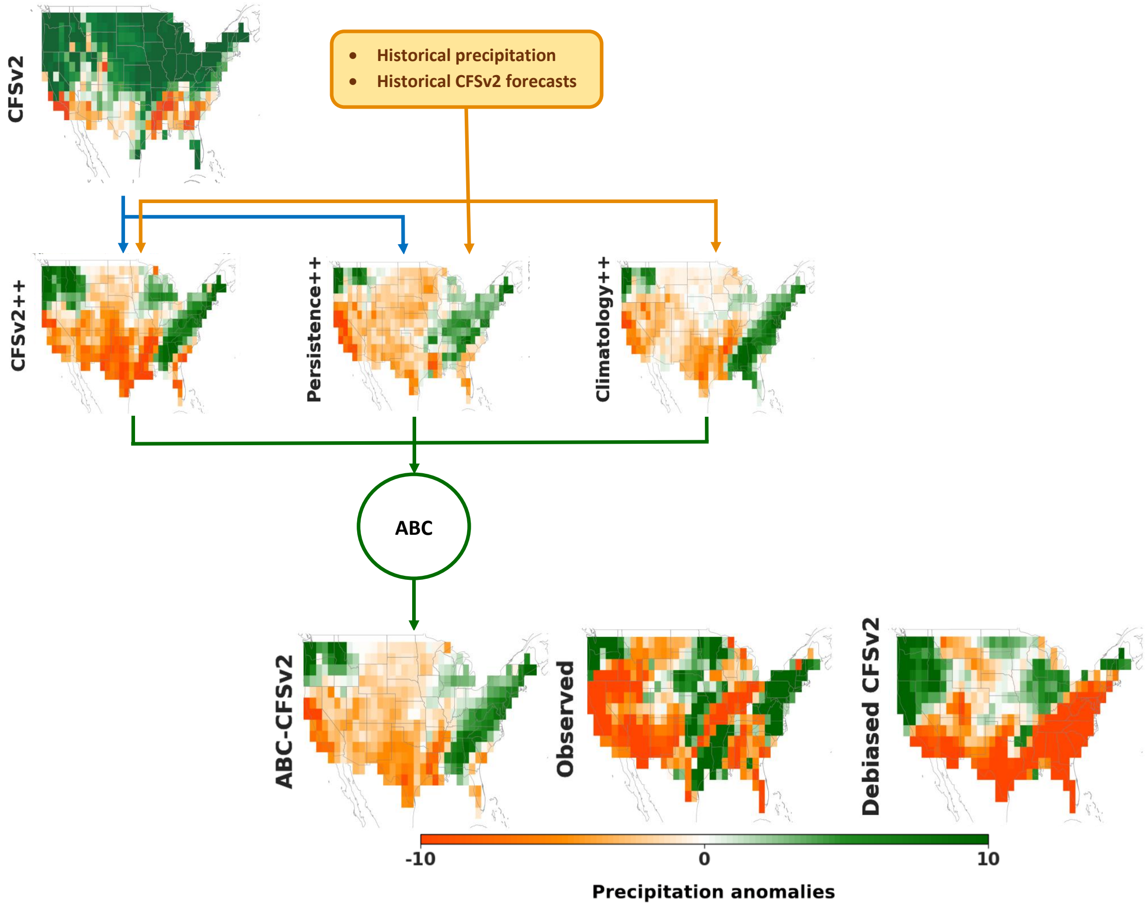

Figure S15: **Schematic of adaptive bias correction (ABC) model input and output data for weeks 3-4 precipitation forecasting.** Here, we compare ABC-corrected CFSv2 forecast with operationally debiased CFSv2 and the ground-truth observations for the target date 2020-12-18.

B Supplementary Methods

B.1 ABC algorithm details

This section presents the algorithm details for the three machine learning models underlying ABC: Dynamical++, Climatology++, and Persistence++.

Algorithm S1 Dynamical++

input test date $t^\star$; lead time $l^\star$; # issuance dates $d^\star$; span s; training set ground truth and dynamical forecasts $(\mathbf{y}_t, \mathbf{f}_{t,l})_{t\in\mathcal{T},l\in\mathcal{L}}$
initialize days per year $D = 365.242199$; # training years $Y = 12$
$\mathcal{S} = \{t \in \mathcal{T} : \texttt{year\_diff} \coloneqq \lfloor \frac{t^\star - t}{D} \rfloor \leq Y$ and $\texttt{day\_diff} \coloneqq \frac{365}{2} - ||(t^\star - t) \mod D| - \frac{365}{2}| \leq s\}$
// Form dynamical ensemble forecast across issuance dates and lead times $l \in \mathcal{L}$
for training and test dates $t \in \mathcal{S} \cup \{t^\star\}$ **do**
$\bar{\mathbf{f}}_t = \text{mean}((\mathbf{f}_{t-l^\star-d+1,l})_{1\leq d\leq d^\star, l\in\mathcal{L}})$
output $\bar{\mathbf{f}}_{t^\star} + \text{mean}((\mathbf{y}_t - \bar{\mathbf{f}}_t)_{t\in\mathcal{S}})$

Algorithm S2 Climatology++

input test date $t^\star$; # train years Y; span s; loss $\in \{\text{RMSE,MSE}\}$; training set ground truth $(\mathbf{y}_t)_{t\in\mathcal{T}}$
initialize days per year $D = 365.242199$
$\mathcal{S} = \{t \in \mathcal{T} : \texttt{year\_diff} \coloneqq \lfloor \frac{t^\star - t}{D} \rfloor \leq Y$ and $\texttt{day\_diff} \coloneqq \frac{365}{2} - ||(t^\star - t) \mod D| - \frac{365}{2}| \leq s\}$
output $\text{argmin}_{\mathbf{y}} \sum_{t\in\mathcal{S}} \text{loss}(\mathbf{y}, \mathbf{y}_t)$

Algorithm S3 Persistence++

input lead time $l^\star$; training set ground truth, climatology, and dynamical forecasts $(\mathbf{y}_t, \mathbf{c}_t, \mathbf{f}_{t,l})_{t\in\mathcal{T},l\in\mathcal{L}}$
initialize forecast period length $L = 14$
// Form dynamical ensemble forecast across subseasonal lead times $l \geq l^\star$
for training dates $t \in \mathcal{T}$ **do**
$\bar{\mathbf{f}}_t = \text{mean}((\mathbf{f}_{t,l})_{l\geq l^\star})$
// Combine ensemble forecast, climatology, and lagged measurements
for grid points $g = 1$ **to** G **do**
$\hat{\boldsymbol{\beta}}_g \in \text{argmin}_{\boldsymbol{\beta}} \sum_{t\in\mathcal{T}} (y_{t,g} - \boldsymbol{\beta}^\top [1, c_{t,g}, y_{t-l^\star-L-1,g}, y_{t-2l^\star-L-1,g}, \bar{f}_{t-l^\star-1,g}])^2$
output coefficients $(\hat{\boldsymbol{\beta}}_g)_{g=1}^G$

B.2 Probabilistic evaluation

ABC probabilistic forecast

To construct a probabilistic forecast from the deterministic outputs of ABC, we first apply the Dynamical++, Climatology++, and Persistence++ models to the dynamical model ensemble mean to generate deterministic forecasts as usual. We then bias correct the forecast of each dynamical model ensemble member by adding the Dynamical++ deterministic forecast and subtracting the raw dynamical model ensemble mean forecast. We then repeat this process using Persistence++ for bias correction in place of Dynamical++. After these bias correction steps, any negative forecasted precipitation values are replaced with 0. Finally, we use the

empirical distribution over Dynamical++-corrected ensemble members, Persistence++-corrected ensemble members, and Climatology++ as our probabilistic forecast distribution. In the case of ECMWF, there are 51 raw ensemble member forecasts (consisting of a single control forecast and 50 perturbed forecasts), so the associated ABC-ECMWF probabilistic forecast is an empirical distribution over 103 corrections.

Baseline probabilistic forecasts

To construct a probabilistic forecast from the deterministic output of a baseline debiasing procedure (like LOESS or quantile mapping), we first apply the debiasing procedure to the dynamical model ensemble mean to generate deterministic forecasts as usual. We then bias correct the forecast of each dynamical model ensemble member by adding the baseline deterministic forecast and subtracting the raw dynamical model ensemble mean forecast. After this bias correction step, any negative forecasted precipitation values are replaced with 0. Finally, we use the empirical distribution over the corrected ensemble members as our probabilistic forecast distribution.

Continuous ranked probability score

For each target date t and grid point g, the continuous ranked probability score (CRPS) [1] is measured as

$$\mathrm{CRPS}(\hat{F}_{t,g}, y_{t,g}) = \int_{-\infty}^{\infty} (\hat{F}_{t,g}(x) - \mathbb{I}[y_{t,g} \leq x])^2 dx \tag{S1}$$

where $y_{t,g}$ represents the ground-truth observation for the date and grid point and $\hat{F}_{t,g}$ represents the predicted cumulative distribution function for the date and grid point. A smaller CRPS value indicates a higher quality probabilistic forecast.

Brier skill score

For each target date t, grid point g, and user-supplied threshold $x_{t,g}$, the Brier score (BS) [1] is measured as

$$\mathrm{BS}(\hat{F}_{t,g}, y_{t,g}; x_{t,g}) = (\hat{F}_{t,g}(x_{t,g}) - \mathbb{I}[y_{t,g} \leq x_{t,g}])^2 \tag{S2}$$

where $y_{t,g}$ represents the ground-truth observation for the date and grid point and $\hat{F}_{t,g}$ represents the predicted cumulative distribution function for the date and grid point. For the threshold $x_{t,g}$, we choose the second tercile of the climatological distribution of observations from the same grid point, day, and month in the years 1981–2010, so that the BS equivalently measures the error of estimating the probability of above-normal (versus near-normal or below-normal) observations. For this choice of thresholds, the Brier skill score (BSS) [1], which compares the the average BS of a forecast to the average BS of climatology across all grid points, takes the form

$$\mathrm{BSS}(\hat{\boldsymbol{F}}_t, \mathbf{y}_t; \boldsymbol{x}_t) = 1 - \frac{\frac{1}{G}\sum_{g=1}^{g} \mathrm{BS}(\hat{F}_{t,g}, y_{t,g}; x_{t,g})}{\frac{1}{G}\sum_{g=1}^{g} (\frac{2}{3} - \mathbb{I}[y_{t,g} \leq x_{t,g}])^2} \tag{S3}$$

for $\mathbf{y}_t \in \mathbb{R}^G$ the vector of ground-truth observations, $\boldsymbol{x}_t \in \mathbb{R}^G$ the vector of thresholds, and $\hat{\boldsymbol{F}}_t$ the collection of predicted cumulative distribution functions for each grid point. A larger BSS value indicates a higher quality probabilistic forecast.

Supplementary References

[1] D. S. Wilks, *Statistical Methods in the Atmospheric Sciences*, ser. International Geophysics. Academic Press, 2011, vol. 100.
